# Assessment of Chemical and Physico-Chemical Properties of Cyanobacterial Lipids for Biodiesel Production

**DOI:** 10.3390/md11072365

**Published:** 2013-07-04

**Authors:** Patrícia C. M. Da Rós, Caroline S. P. Silva, Maria E. Silva-Stenico, Marli F. Fiore, Heizir F. De Castro

**Affiliations:** 1 Engineering School of Lorena, University of São Paulo, 12602-810, Lorena SP, Brazil; E-Mails: carolpamplona@gmail.com (C.S.P.S.); heizir@dequi.eel.usp.br (H.F.C.); 2 Center of Nuclear Energy in Agriculture, University of São Paulo, 13400-970, Piracicaba SP, Brazil; E-Mails: estela88@hotmail.com (M.E.S.-S.); fiore@cena.usp.br (M.F.F.)

**Keywords:** fatty acid, lipid feedstock, productivity, cyanobacteria, biofuel

## Abstract

Five non-toxin producing cyanobacterial isolates from the genera *Synechococcus*, *Trichormus*, *Microcystis*, *Leptolyngbya* and *Chlorogloea* were examined in terms of quantity and quality as lipid feedstock for biofuel production. Under the conditions used in this study, the biomass productivity ranged from 3.7 to 52.7 mg·L^−1^·day^−1^ in relation to dry biomass, while the lipid productivity varied between 0.8 and 14.2 mg·L^−1^·day^−1^. All cyanobacterial strains evaluated yielded lipids with similar fatty acid composition to those present in the seed oils successfully used for biodiesel synthesis. However, by combining biomass and lipid productivity parameters, the greatest potential was found for *Synechococcus* sp. PCC7942, *M. aeruginosa* NPCD-1 and *Trichormus* sp. CENA77. The chosen lipid samples were further characterized using Fourier Transform Infrared spectroscopy (FTIR), viscosity and thermogravimetry and used as lipid feedstock for biodiesel synthesis by heterogeneous catalysis.

## 1. Introduction

Cyanobacteria are a large group of oxygenic photoautotrophic bacteria and, like plants and algae, can capture CO_2_ via the Calvin-Benson cycle and convert it to a suite of organic compounds. They are important primary producers of organic material and play significant roles in biochemical cycles of carbon, nitrogen, and oxygen [1]. Cyanobacteria are well suited for synthetic biology and metabolic engineering approaches for the phototrophic production of various desirable biomolecules, including substances with cytotoxic, antifungal, antibacterial and antiviral activities [2,3]. Lipids, carotenoids, pigments, vitamins and aromatic compounds are also found in cyanobacteria. Lipids (accumulated in the thylakoid membranes) are associated with high levels of photosynthesis and rapid growth rate and are of particular interest, since they can be used as lipid feedstock for biodiesel production [4,5,6]. Microalgae accumulate large amounts of lipids as reserve material, but only in conditions of stress and slow growth [7]. Thus, cyanobacteria have a natural advantage to produce lipids in high-speed growth.

Currently, biodiesel derived from microbial biomass is attracting much attention and investment and can meet the demand necessary for biodiesel supply without commitment to the food chain. The biosynthesis of fatty acid-based biofuels in cyanobacteria includes two steps, production and transesterification of fatty acids (FAs) to form alkyl FA esters [8]. Considering that fuel properties are largely dependent on the FA composition of the feedstock from which biodiesel is prepared, FA profile was employed as a screening tool for selection of cyanobacterial lipids with high amounts of monounsaturated FAs. The presence of double bonds in the FAs from cyanobacterial lipids is related to their morphological complexity [9]. It has been reported that unicellular forms are characterized by the presence of mono and polyenoic acids [10].

In order to compare representatives of four different cyanobacterial orders [11], five strains isolated from extreme biomes were chosen: *Synechococcus* sp. PCC7942 (Synechococcales); *Microcystis aeruginosa* NPCD-1 and *Chlorogloea* sp. CENA170 (Chroococcales), isolated from a sewage treatment plant and the mangrove, respectively; *Leptolyngbya* sp. CENA104 (Pseudanabaenales), which is classified as a filamentous cyanobacteria without heterocytes, also isolated from a sewage treatment plant; *Trichormus* sp. CENA77 (Nostocales), a heterocystous filamentous strain, isolated from a contaminated environment containing the herbicide propanil.

In addition, three of them are unicellular strains (*Synechococcus* sp. PCC7942, *M. aeruginosa* NPCD-1 and *Chlorogloea* sp. CENA170), which allow easy genetic manipulation in order to increase the photosynthetic efficiency, growth rate and lipid content in the biomass [12]. On the other hand, the two filamentous strains (*Leptolyngbya* sp. CENA104 and *Trichormus* sp. CENA77) have the advantage of easy procedures for biomass harvesting [13].

Although cyanobacteria have several advantages for biofuel production, including easy genetic manipulation to increase photosynthetic efficiency, fast growth rate and high lipid content in the biomass, there are few specific studies on their use as a source of lipids. In this context, this study aims to evaluate the potential of five cyanobacterial strains as producers of lipid feedstock for the synthesis of biodiesel. Biomass and lipid productivities and FA profiles were evaluated. Other selection criteria included were iodine value, degree of unsaturation and long chain saturated factor. 

## 2. Results and Discussion

### 2.1. Biomass and Lipid Productivity

The biomass productivity ranged from 3.7 to 52.7 mg·L^−1^·day^−1^ in relation to dry biomass while the lipid productivity varied between 0.8 and 14.2 mg·L^−1^·day^−1^ (Table 1). The highest lipid productivity was obtained by *Synechococcus* sp. PCC7942 and the lowest by *Leptolyngbya* sp. CENA104. High biomass and lipid productivities were also found for *M. aeruginosa* NPCD-1 (46.9 and 13.1 mg·L^−1^·day^−1^, respectively) and *Trichormus* sp. CENA77 (30.8 and 7.3 mg·L^−1^·day^−1^, respectively). The biomass from *Chlorogloea* sp. CENA170 and *Leptolyngbya* sp. CENA104 attained low lipid productivity (lower than 1.0 mg·L^−1^·day^−1^) and were considered unsuitable as a source of lipid feedstock.

**Table 1:** Cyanobacterial strains used in this study and their respective biomass and lipid productivity.

Cyanobacterium	Micrograph	Strain source	Biomass productivity	Lipid productivity
	Scale bar: 20 µm		(mg·L^−1^·day^−1^)	(mg·L^−1^·day^−1^)
*Microcystis aeruginosa* NPCD-1	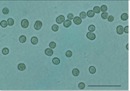	Sewage treatment plant of Cidade de Deus, RJ, Brazil	46.9	13.1
*Synechococcus* sp. PCC7942	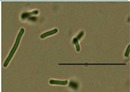	Pasteur Culture Collection, France	52.7	14.2
*Chlorogloea* sp. CENA170	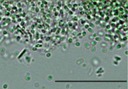	Mangrove soil from Cardoso Island, SP, Brazil	6.8	0.9
*Trichormus* sp. CENA77	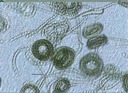	Flooded rice field, SC, Brazil	30.8	7.3
*Leptolyngbya* sp. CENA104	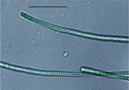	Sewage treatment plant of Cajati, SP, Brazil	3.7	0.8

For the three selected isolates, lipid content (28.0% for *M. aeruginosa* NPCD-1, 26.9% for *Synechococcus* sp. PCC7942 and 23.7% for *Trichormus* sp. CENA77) were similar to those reported in a study in which 13 different species of cyanobacteria isolated from aquatic habitats showed lipid content between 10.5 and 28.1 wt% [14]. The highest lipid content (28.1%) was obtained by *M. aeruginosa* followed by *Phormidium purpurascens* (26.4%), while *P. ambiguum* had the lowest value.

Another study showed that biomass productivity attained by *Aphanotece microscopic* Nägeli was 753.6 mg·L^−1^·day^−1^, but lipid content was low as 8.0 wt% [15]. The microalgae *Scenedesmus* sp. yielded 220 mg·L^−1^·day^−1^ of biomass productivity and 9.5 wt% of lipid content [16], whereas the cyanobacterium *Spirulina maxima* LB 2342 presented biomass productivity of 210 mg·L^−1^·day^−1^ and 4.1% of lipid content [17]. *Scenedesmus obliquus* sp. resulted in a biomass productivity of 60 mg·L^−1^·day^−1^ and 12.7% of lipids [18]. 

The lipid content of cyanobacteria can range from 5 to 13 wt% [19]. However, other authors reveal that the lipid content accumulated by cyanobacteria can vary from 38 to 45 wt%, depending on the species and environmental conditions, including nutrients and light intensity [4,20]. Thus, the results of lipid content (from 13% to 28%) obtained in the present study for the assessed species are favorable compared to those reported for other species and genera of cyanobacteria.

### 2.2. Fatty Acids Profile

Besides the favorable lipid productivity, the selected strains should have a FA profile that allows obtaining biodiesel with the physico-chemical properties required to be used as a fuel. The FA profile of cyanobacterial lipid characterized by GC yielded 21 FAs with carbon chains ranging from C6 to C24 and different degrees of unsaturation (Table 2). The percentage of unidentified FAs was approximately 1%, with the exception of lipid biomass from *M. aeruginosa* NPCD-1, which showed 3.4% of unidentified FAs.

**Table 2:** Fatty acid (FA) profile (% wt) present in the cyanobacterial lipid.

FA		*Microcystis* *aeruginosa* NPCD-1	*Synechococcus* sp. PCC7942	*Chlorogloea* sp. CENA170	*Trichormus* sp. CENA77	*Leptolyngbya* sp. CENA104
*Saturated FA (%wt)*						
C6:0	Caproic	0.1	0.06	0.05	0.08	0.09
C8:0	Caprilic	0.6	0.06	0.4	0.6	0.2
C10:0	Capric	0.9	0.06	0.6	0.6	0.2
C12:0	Lauric	13.2	0.7	8.7	9.7	3.9
C14:0	Myristic	5.2	0.7	3.4	3.9	1.7
C15:0	Pentadecanoic	0.1	0.06	0.06	0.06	0.08
C16:0	Palmitic	24.3	23.5	15.4	24.9	14.6
C17:0	Margaric	0.2	0.1	0.1	0.2	0.15
C18:0	Stearic	4.9	3.6	3.8	3.4	2.8
C20:0	Arachidic	0.3	0.3	0.2	0.37	0.4
C22:0	Behenic	0.2	0.3	0.2	0.15	0.3
C24:0	Lignoceric	0.1	0.2	0.1	0.10	0.15
	*Total*	*50.1*	*29.6*	*33.0*	*44.1*	*24.6*
*Monounsaturated* *FA*						
C16:1	Palmitoleic	1.1	3.3	5.4	1.5	3.9
C17:1	*cis*-10-Heptadecenoic	0.1	0.1	0.1	0.1	0.2
C18:1	Oleic	26.8	31.5	28.8	36.9	38.8
C20:1	Gadoleic	0.3	0.2	0.1	0.3	0.42
C22:1	Erucic	2.5	nd	nd	nd	nd
C24:1	Nervonic	0.8	nd	nd	nd	nd
	*Total*	*31.6*	*35.1*	*34.4*	*38.8*	*43.3*
*Polyunsaturated FA*						
C18:2	Linoleic	12.5	30.9	29.4	10.7	26.4
C18:3	.-Linolenic	0.8	0.5	0.27	nd	0.4
C18:3	Linolenic	1.6	2.9	1.63	5.1	4.3
	*Total*	*14.9*	*34.3*	*31.3*	*15.8*	*31.1*
N.I.	*Unidentified*	3.4	1.0	1.3	1.3	1.0

n.d. = not detected.

Most FAs present in the five cyanobacterial isolates studied were unsaturated (46%–74%) although saturated FAs were also found (24%–50%). The majority FA were palmitic acid (C16:0), ranging from 14% to 25%, and oleic (C18:1) and linoleic (C18:2) acids, ranging from 26% to 38% and from 10% to 30%, respectively. 

*M. aeruginosa* NPCD-1 biomass had approximately 50% of saturated FAs, with a higher proportion of palmitic (24.3%) and lauric (13.2%) acids. Unsaturated FAs obtained were 31% of monounsaturated and 14.9% polyunsaturated, with a higher proportion of oleic (C18:1) and linoleic (C18:2) acids, which represented 26.8% and 12.5%, respectively. A similar profile was obtained for *Trichormus* sp. CENA77 biomass, which contained 44.1% of saturated and 54.6% of unsaturated FAs. Oleic acid was present in a higher proportion (36.9%), followed by palmitic (24.9%), linoleic (10.7%) and lauric (9.7%) acids.

*Synechococcus* sp. PCC7942 showed 29.6% of saturated FAs and 69.4% of unsaturated FAs, with a greater proportion of oleic (31.5%), linoleic (30.9%) and palmitic (23.5%) acids. *Chlorogloea* sp. CENA170 biomass had 33% of saturated FAs (palmitic = 15.4% and lauric = 8.7%) and 65.7% of unsaturated FAs, including linoleic (29.4%) and oleic (28.8%) acids in the higher proportion. The lipid from *Leptolyngbya* sp. CENA104 presented 24.6% of saturated and 74.4% of unsaturated FAs, with oleic (38.8%) and linoleic (26.4%) acids in greater proportion, followed by palmitic acid (14.6%).

These results confirm previously reported data regarding the ability of cyanobacteria to synthesize simpler FAs than eukaryotic microalgae [20]. It can be noticed that all tested strains produced high amounts of FAs, such as palmitic (C16-saturated), oleic (C18:1-monounsaturated) and linoleic (C18:2-polyunsaturated) acids. All cyanobacteria strains evaluated in this work showed a FA profile similar to the seed oils successfully used as lipid feedstocks for biodiesel synthesis [21,22].

Biodiesel is produced by reacting a short chain alcohol with biologically derived oils that consist of FAs ester-linked to a glycerol molecule. Considering that the FAs themselves are not modified by the reaction, the fatty esters composition of the fuel is directly equivalent to the FAs composition of the biomass feedstock. The fatty esters composition determines the fuel properties [23] and the most important attributes that affect the fuel properties are the length of the carbon chain and the number of double bonds. These also affected the viscosity of biodiesel, which is about an order of magnitude lower than that of the parent oil and depends on the composition of alkyl esters [24]. High viscosity is the major fuel property why neat vegetable oils have been largely abandoned as alternative diesel fuel. Viscosity increases with chain length (number of carbon atoms) and with increasing degree of saturation. This holds also for the alcohol moiety as the viscosity of ethyl esters is slightly higher than that of methyl esters [23].

### 2.3. Cyanobacterial Fatty Acid Composition: Implications for Biodiesel Quality

Using data on the content of individual FAs, the quality of biodiesel could be predicted by calculating IV (iodine value), SV (Saponification value), DU (Degree of unsaturation) and LCSF (long chain saturated factor) using empirical equations described by Ramos *et al.* [24] as displayed in Table 3.

**Table 3:** Properties of lipid feedstock from cyanobacteria.

Property	*M. aeruginosa* NPCD-1	*Synechococcus* sp. PCC7942	*Chlorogloea* sp. CENA170	*Trichormus* sp. CENA77	*Leptolyngbya* sp. CENA104
Iodine value (g I_2_/100 g)	57	97	90	68	100
Saponification value	210	203	210	213	205
Degree of unsaturation (%)	60.7	103.7	97	70.4	105.5
Long chain saturated factor	5.7	5.2	4.1	5.0	4.0

Values for IV ranged from 57 to 100 g I_2_/100 g, which are in accordance with European Standard UNE-EN 14214 [25] Lipids from *M. aeruginosa* NPCD-1, which yielded approximately 50% of saturated FAs and 46% of unsaturated FAs, resulted in an IV of 57 g I_2_/100 g and a DU equal to 60.7%. Similar results were obtained for lipids from *Trichormus* sp. CENA77, which resulted in an IV of 68 g I_2_/100 g and a DU of 70.4%. All of these parameters comply with the limits established by US Standard (ASTM D6751-02) [26], European Standard (EN 14214) [25] and Brazilian National Petroleum Agency Standard (ANP Resolution N° 14/2012) [27] for biodiesel quality.

LCSF of lipid feedstock is a critical parameter for oxidation stability, cetane number, IV and cold filter plugging point (CFPP) of the biodiesel obtained. Some authors reported that the longer the biodiesel carbon chains, the worse their low-temperature properties. In addition, when a liquid biodiesel is cooled, the alkyl esters of stearic and palmitic acids are the first to precipitate and, therefore, typically constitute a major share of material recovered from clogged biodiesel fuel filters [15].

Lipids from *M. aeruginosa* NPCD-1, *Synechococcus* sp. PCC7942 and *Trichormus* sp. CENA77 have the highest LCSF, since they contain a higher concentration of palmitic and stearic FAs (Table 2).

Low-temperature properties depend mostly on the saturated ester content and the effect of unsaturated ester composition can be considered negligible. In this sense, the CFPP of the biodiesel is correlated with the LCSF [24].

As shown by the FA profile, IV, SV, DU and LCSF, all tested strains are promising sources of lipid feedstock for biodiesel production, as well as satisfying biodiesel quality standards. However, *Synechococcus* sp. PCC7942, *M. aeruginosa* NPCD-1 and *Trichormus* sp. CENA77 had better properties as feedstock for biodiesel production. The highest values for biomass and lipid productivity were attained by *Synechococcus* sp. PCC7942 (52.7 and 14.2 mg·L^−1^·day^−1^, respectively) and *M. aeruginosa* NPCD-1 (46.9 and 13.1 mg·L^−1^·day^−1^, respectively) followed by *Trichormus* sp. CENA77 (30.8 and 7.3 mg·L^−1^·day^−1^, respectively).

### 2.4. Characterization of Lipid Materials

**Figure 1:**
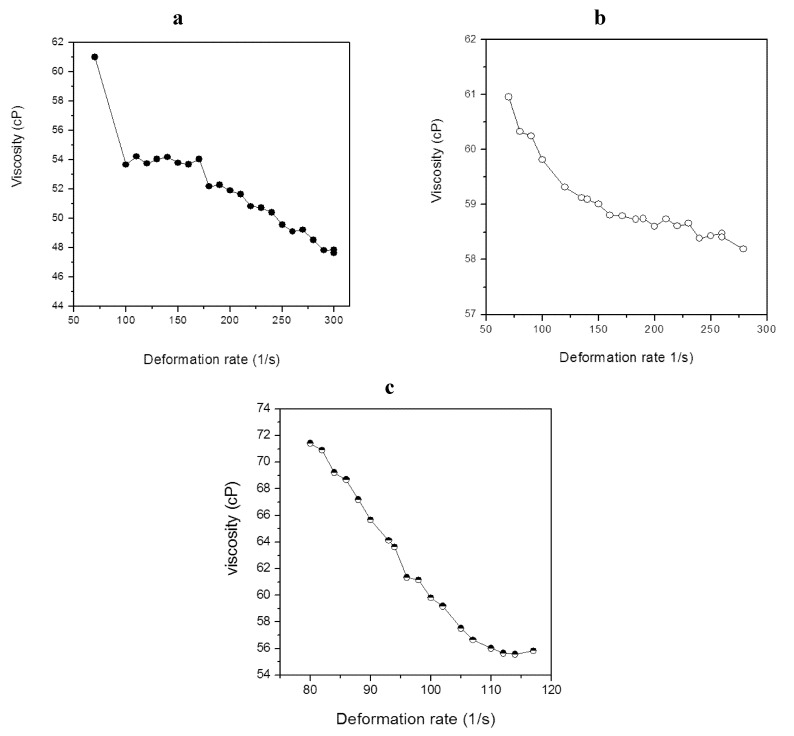
Absolute viscosity as function of shear rate for cyanobacterial lipid from *M. aeruginosa* NPCD-1 (**a**), *Trichormus* sp. CENA77 (**b**) and *Synechococcus* sp. PCC7942 (**c**).

Rheological studies concerning the behavior and properties of lipid materials can be very useful in the design and evaluation of equipment used for the development of lipid-based diesel fuel substitutes derived from microbial oils. In this context the lipid from the selected cyanobacterial isolates were evaluated to verify the rheological behavior of the liquid oils. Figure 1 (a,b,c) shows the change in viscosity as a function of shear rate for the behavior of the absolute viscosity of the lipid material at 50 °C. The amount of viscosity (cP) decreases as the shear rate increase, characterizing the behavior of the non-Newtonian liquid oil. The analysis of the curve allowed an adjustment of the shear stress as a function of shear rate according to the power law.

Table 4 shows the adjustment coefficients for the log t data as a function of log γ [28]. For all lipids analyzed, the value of the angular coefficient was lower than 1, characterizing them as pseudoplastic fluids, where the viscosity decreases with an increasing shear rate [28].

**Table 4:** Values of viscosity coefficient and adjustment of power law [28] data.

Strain	Viscosity (cP)	η *
*M. aeruginosa* NPCD-1	52.7	0.84
*Trichormus* sp. CENA77	59.1	0.97
*Synechococcus* sp. PCC7942	62.3	0.37

* Calculated according to equation 5 at 50 °C described at the experimental section (item 3.4).

Within the applied range of deformation (70 to 300 s^−1^), the lipid materials from *M. aeruginosa* NPCD-1 and *Trichormus* sp. CENA77 showed absolute viscosity average of 52.7 cP and 59.1 cP at 50 °C, respectively, while lipid from *Synechococcus* sp. PCC7942 biomass showed 62.3 cP (deformation range between 70 and 120 s^−1^). When comparing these values with the viscosity of traditional feedstocks, such as palm oil (31.1 cP) and beef tallow (43.8 cP), they present a different rheological behavior, since vegetable oils and residual fat generally behave as a Newtonian fluid [21,22].

Biodiesel synthesis from high viscosity lipids such as beef tallow, for example, results in viscosity values that meet the fuel standards [29]. Therefore, it can be predicted that the high viscosity of the lipid biomass is not a limiting parameter for dimensioning reactors to produce biodiesel. However, it is a limitation that should be considered and that requires the use of solvents in the reaction, driven mainly by the non-Newtonian fluid behavior. Previous work carried out by our group [12] has demonstrated that the use of iso-octane as solvent allowed almost full conversion of *M. aeruginosa* NPCD-1 lipid (52.7 cP) into ethyl esters by enzymatic route. 

Thermal analysis (TGA) generates a curve of thermal decomposition that gives the degradation stages of the samples as a function of temperature [30]. By analyzing these data, it is possible to establish parameters for the thermal stability of oils and fats and compare it to the stability of biodiesel. Furthermore, the TGA has been used to study the behavior of biomass combustion [31].

**Figure 2:**
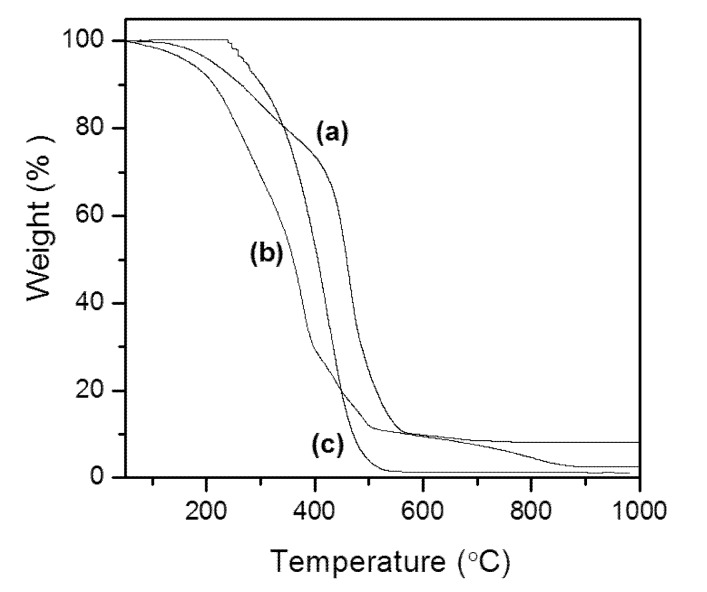
Thermal study (TGA) for cyanobacterial lipid from *M. aeruginosa* NPCD-1 (**a**), *Trichormus* sp. CENA77 (**b**) and *Synechococcus* sp. PCC7942 (**c**).

Figure 2 displays the curves of TG of the lipid materials, which demonstrate the percentage of mass loss versus temperature. The mass losses from *M. aeruginosa* NPCD-1, *Trichormus* sp. CENA77 and *Synechococcus* sp. PCC7942 show only one stage of thermal degradation that began at about 280, 200 and 250 °C, respectively, and extended to approximately 500, 400 and 430 °C, respectively. These steps relate to the decomposition and volatilization of lipid present in the samples (triglycerides and free FAs). During this process, the temperature of maximum mass loss observed by the TG curve occurred at 497 °C for *M. aeruginosa* NPCD-1, 380 °C for *Trichormus* sp. CENA77 and 405 °C for *Synechococcus* sp. PCC7942.

These results corroborate to those reported by Carvalho [32], in which various traditional feedstocks, such as andiroba, babassu, jatropha oils and beef tallow were evaluated by TGA. The results also indicated a similarity between the intervals at which mass loss occurs for babassu and andiroba oils and beef tallow: 200 to 321 °C, 321 to 442 °C and 210 to 560 °C, respectively. However, the initial temperature of mass loss for andiroba oil was much lower, with a range between 97 and 376 °C [32]. 

**Figure 3:**
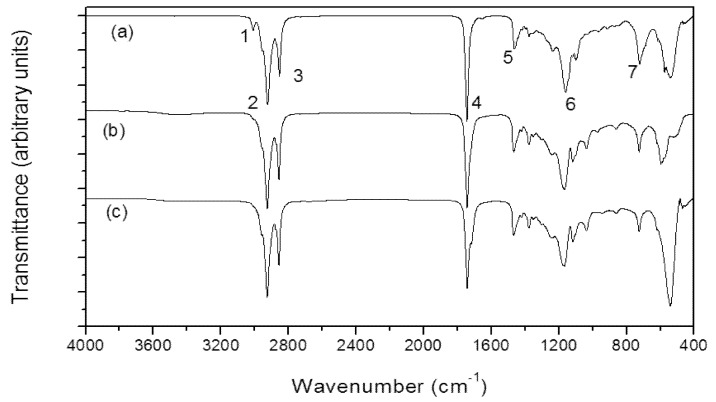
Infrared spectra for cyanobacterial lipid from *M. aeruginosa* NPCD-1 (**a**), *Trichormus* sp. CENA77 (**b**) and *Synechococcus* sp. PCC7942 (**c**).

The use of FT-IR became a powerful tool in lipids analysis, since other analytical methods can be time-consuming. The FAs identification can be assessed through this methodology by the correlation with other analytical data, as used in this study which correlated the IR spectroscopy results with GC. This procedure is a way of cross-checking the results. The measurement is performed by infrared absorption according to a range of molecular vibrational modes [33]. The contents and functional groups of the lipid extracts from cyanobacterial biomass were observed between 400 and 4000 cm^−1^. The spectra of the cyanobacterial lipid from *M. aeruginosa* NPCD-1, *Synechococcus* sp. PCC7942 and *Trichormus* sp. CENA77 are displayed in Figure 3. The assignments for the infrared bands were based on the correlations with FAs, esters and triglycerides of FAs and similar results were found for andiroba oil as displayed in Table 5.

**Table 5:** Assignment of principal infrared bands identified by FT-IR spectroscopy to evaluate cyanobacterial lipids.

Band	Assignment	Lipid from cyanobacteria (this study)	Andiroba oil (cm^−1^) *
1	(=C–H) stretching	3006	3005
2	CH_2_ asymmetry stretching	2928	2923
3	CH_2_ symmetry stretching	2852	2854
4	C=O stretching	1744	1741
5	CH_2_ scissors	1464	1463
6	C=C–C–O	1150	1040–1290
7	CH_2_ rocking	724	722

* Reference [33].

All samples showed a spectrum with vibration band at 3006 cm^−1^ related to the CH stretching of =C–H bonding (band 1). The two intense bands at 2928 cm^−1^ (band 2) and 2852 cm^−1^ (band 3) are due to CH_2_ asymmetric and symmetric stretching vibrations, respectively. These vibrations were also observed at 2926 and 2855 cm^−1^ for the spectra of several vegetal oils such as soybean, palm and palm kernel oils [34]. The band at 1744 cm^−1^ (band 4) is assigned to the C=O stretching vibration of the carboxylic groups, and occurs in the range of 1750–1735 cm^−1^ [34,35,36]. It appears around the same frequency for methyl esters and triglycerides; for long-chain FAs it appears at around 1700 cm^−1^.

Hence, the observation of this band in the spectrum of the cyanobacterial lipids from *M. aeruginosa* NPCD-1, *Synechococcus* sp. PCC7942 and *Trichormus* sp. CENA77 indicates the presence of the ester function. At 1464 cm^–1^ a band 5 was observed, which is assigned to CH_2_ scissors deformation vibration [34]. This vibration was observed as a single band at 1475 cm^−1^. The band at 1150 cm^−1^ (band 6) is due to C–O (esters), stretch in two or more bands, one stronger and broader than the other, occurred in the range 1300–1000 cm^−1^ [33]. The band at 726 cm^−1^ (band 7) observed in the spectrum is assigned to the CH_2_ rocking mode [34]. The bending motion associated with four or more CH_2_ groups in an open chain and occurs at about 724 cm^−1^ (called a long-chain band) [36].

### 2.5. Ethanolysis of Lipid Feedstocks

**Figure 4:**
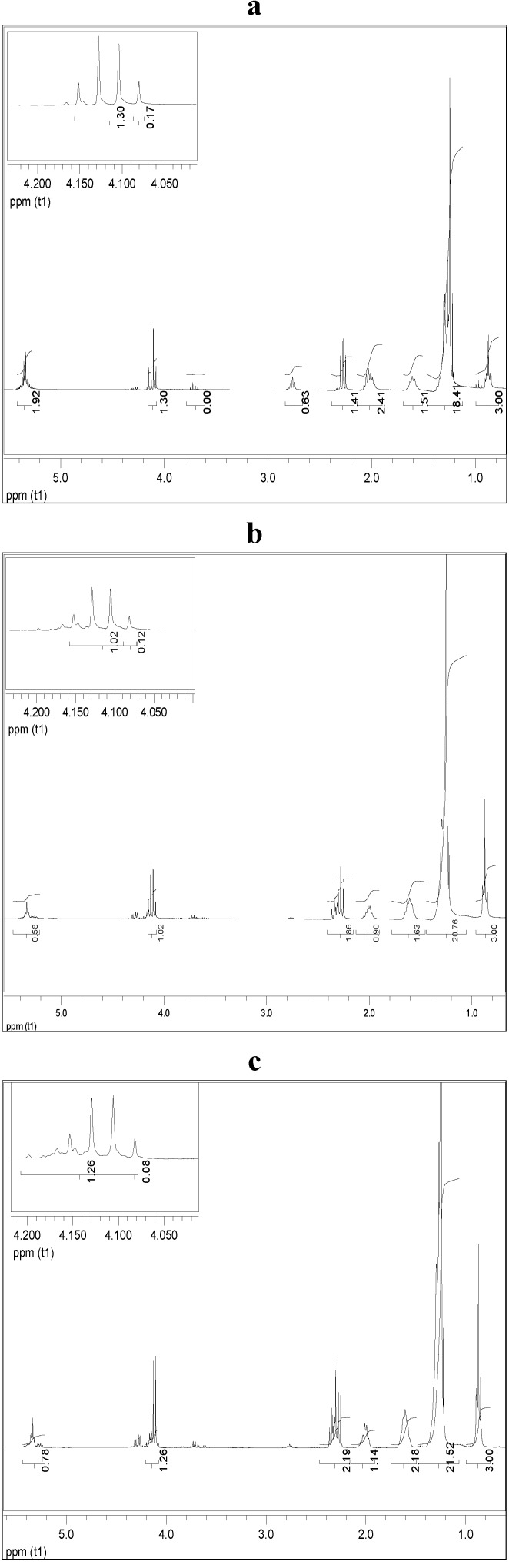
^1^H NMR spectra for purified biodiesel from *M. aeruginosa* NPCD-1 (**a**), *Synechococcus* sp. PCC7942 (**b**) and *Trichormus* sp. CENA77 (**c**).

The feasibility of the selected cyanobacteria lipid to yield biodiesel using ethanol as acyl acceptor was performed using niobium impregnated with NaOH as catalyst. This procedure was based on the efficiency of this catalyst to carry out transesterification reactions of several vegetal oils [32]. Experiments were conducted under a preliminary set of reaction conditions (oil-to-ethanol proportion of 1:4; 78.5 °C and 10% of catalyst) that may not have been the optimum set for all the feedstocks. Biodiesel samples were quantified by ^1^H NMR, which allowed evaluating the conversion of raw materials into biodiesel, based on the monitoring of changes in the sign of the hydrogens of glycerin, thus enabling confirm the occurrence of the transesterification reaction of triglycerides and its conversion into ethyl esters. Figure 4 shows the ^1^H NMR spectra related to the purified biodiesel samples from *M. aeruginosa* NPCD-1 (a), *Synechococcus* sp. PCC7942 (b) and *Trichormus* sp. CENA77 (c). 

In the spectra corresponding to the ethanolysis of lipid material of *M. aeruginosa* NPCD-1 (Figure 4a), multiples at 4.10–4.32 ppm, attributed to the protons on the 1 and 3 carbon atoms of the glycerol group are totally absent due to the conversion of triglycerides into ethyl esters. This also shows that the formation of ethyl esters occurred at the region from 4 to 4.2 ppm, being represented by a quartet. This spectrum provided evidence that a complete transesterification reaction had occurred (100%). The ethanolysis from lipid material of *Synechococcus* sp. PCC7942 provided a similar conversion (94.2%) as shown in spectrum (Figure 4b).

On the other hand, much lower conversion (≈50%) was obtained for ethanolysis from lipid material of *Trichormus* sp. CENA77 (Figure 4c) which was associated to the acid value determined as 15.7 mg KOH/g of the sample which corresponded to a level of 9 wt % FFA. Lipid with FFA content higher than 5 wt% is not suitable for base catalyzed transesterification as the FFA will tend to consume the catalyst and form soap which will cause serious separation problem to the product [37]. For this particular case, acid catalyzed transesterification is recommended.

Similar results were reported by Carvalho [32] in the ethanolysis from macaw palm using the same heterogeneous catalyst (Nb/Na). The high acidity present in macaw palm feedstock (16.1 mg KOH/g) resulted in poor transesterification yield (20.7%). 

## 3. Experimental Section

### 3.1. Cyanobacterial Strains and Cultivation

Five cyanobacterial strains from the culture collection of the Laboratory of Molecular Ecology of Cyanobacteria (CENA/USP, São Paulo, Brazil) as follows: *Synechococcus* sp. PCC7942, *Microcystis aeruginosa* NPCD-1, *Chlorogloea* sp. CENA170, *Leptolyngbya* sp. CENA104 and *Trichormus* sp. CENA77 were tested (Table 1). Cyanobacteria were grown in flasks containing 8 L of appropriated culture media for each strain: BG-11 [38], ASM-1 [39] and SWBG-11 [40] and kept under constant aeration, light intensity equivalent to 100 µmol photons m^−2^·s^−1^ and controlled temperature at 24 ± 1 °C. The period of cultivation was from 15 to 30 days depending on the strain studied. Cells were harvested by centrifugation and lyophilized.

### 3.2. Total Lipids Extraction and FA Analysis

Total lipids were extracted using the methodology described by Bligh and Dyer [41]. The total lipids were measured gravimetrically and yields were calculated. The lipid extracted was dried in a rotary evaporator to remove remaining residues of solvent and subsequently dried at 60 °C to constant weight. The total lipids were measured gravimetrically, and then lipids contents and yields were calculated. Analysis of fatty acid composition was performed in a capillary gas chromatography (CGC Agilent 6850 Series GC System) according to AOCS procedure [42]. 

### 3.3. Empirical Parameters

Iodine value (IV), saponification value (SV), degree of unsaturation (DU) and long chain saturated factor (LCSF) were empirically determined according to Equations 1–4 as described by Ramos *et al.* [24].

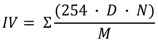
(1)

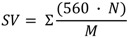
(2)

DU = (wt.% monounsaturated) + 2 × ( wt.% polyunsaturated)
(3)

LCSF = (0.1 × C16) + (0.5 × C18) + (1 × C20) + (1.5 × C22) + (2 × C24)
(4)

### 3.4. Viscosity Determination

The absolute viscosity of lipid was determined with LVDV-II cone and plate spindle Brookfield viscosimeter (Brookfield Viscometers Ltd., Harlow, England) using a CP 52 cone. The shear stress measurements were taken as a function of shear rate and the dynamic viscosity was determined as a constant slope [28]. All measurements were performed in triplicate. The data obtained (viscosity, rate of deformation and shear stress) were adjusted according to the power law (Equation 5) to verify possible deviations from Newtonian behavior [28].

τ = K × γ*^n^* (5)

where: τ is the shear stress, γ is the applied deformation rate, . is the value of angular coefficient and K is the consistency index.

### 3.5. Thermal Study (TG)

TG curves were recorded using thermal balance (TGA-50 Shimadzu thermogravimetric analyzer, Shimadzu, Kyoto, Japan. A dynamic method was used at a heating rate of 10 °C·min^−1^. The initial sample mass was 10.00 ± 0.5 mg, in a nitrogen atmosphere with a flow rate of 50 mL·min^−1^, in a temperature range of 25–1000 °C.

### 3.6. Infrared Spectroscopy (IR)

IR spectra were obtained from a FT-IR Spectrum GX (Perkin Elmer, Perdizes, São Paulo, Brazi) with a spectral resolution of 4 cm^−1^. The spectrum was obtained through the horizontal attenuated total reflectance (HATR) technique using a ZnSe crystal. Spectra in the region 400–4000 cm^−1^ were run and plotted as a percent transmittance curve versus wavenumber.

### 3.7. Biodiesel Synthesis

Biodiesel synthesis from the three selected cyanobacterial biomass were performed in jacket cylindrical glass reactor (6 mm high × 4 mm internal diameter and 150 mL capacity, coupled with a reflux condenser) containing 20 g of substrate (4 g of lipid feedstock and 15 g of ethanol). Niobium oxide impregnated with sodium (Nb_2_O_5_/Na) previously activated in an oven (200 °C for 24 h) was used as catalyst at proportion of 10% in relation to the mass of lipid feedstock. The experiments were carried out at 78.5 °C under mechanical agitation 600 rpm for 10 h [32].

### 3.8. Downstream Procedure

When the reaction was completed the catalyst was separated from the medium and the organic phase was washed twice with one volume of water to remove both the remaining ethanol and the free glycerol as a by-product. The residual ethanol was removed by rotary evaporator to attain the final fatty acid ethyl ester product.

### 3.9. Proton Nuclear Magnetic Resonance Spectrometry (^1^H NMR)

The conversion into ethyl esters was evaluated by ^1^H NMR in a Mercury 300 MHz (Varian spectrometer, Varian, Palo Alto, CA, USA) with 5 mm glass tubes, using CDCl_3_ as the solvent and 0.3% TMS as the internal standard. The calculations involving the conversion of esters were performed using the equation according to methodology described by Paiva and collaborators [43]. This methodology allowed for the identification of molecules by ^1^H NMR, with peaks present in the region from 4.05 to 4.35 ppm during the transesterification reaction.

## 4. Conclusions

*Synechococcus* sp. PCC7942, *M. aeruginosa* NPCD-1 and *Trichormus* sp. CENA77 had the best set of properties to be used as a feedstock source in the synthesis of biodiesel. They showed appropriate values of biomass and lipid productivity, as well as FA profiles similar to the oil seeds already used successfully in the synthesis of biodiesel. Analytical techniques, including infrared spectroscopy, viscosimetry and thermogravimetry showed to be a practical tool to confirm the feasibility of these cyanobacterial lipids for biofuel synthesis. Although further practical investigation regarding the growth of cyanobacterial biomass is required, ethyl esters of cyanobacterial lipids were successfully attained by heterogeneous catalysis.
